# Throttling process of a supersonic cascade studied by high-frequency response pressure and high-speed schlieren

**DOI:** 10.1038/s41598-021-93021-1

**Published:** 2021-06-30

**Authors:** Ziao Wang, Juntao Chang, Wenxin Hou, Daren Yu

**Affiliations:** Harbin Institute of Technology, Harbin, 150001 Heilongjiang People’s Republic of China

**Keywords:** Physics, Fluid dynamics

## Abstract

In this study, a single-channel supersonic cascade model is investigated experimentally at a freestream Mach number of 2.4 to obtain a better understanding of the flow field evolution during the throttling process. A flap is placed at the channel exit to choke the flow linearly. Measurements include 1-kHz schlieren imaging and 10-kHz simultaneous fast-response wall pressure. Three stages, namely attached flow, separated flow, and oscillatory flow, are identified in the throttling process. The joint time–frequency analysis and wall pressure spectrum contour exhibit the time evolution and spatial distribution of the pressure fluctuation. With the increase in backpressure, the pressure fluctuation in the low-frequency shock oscillation range of 40–400 Hz on the suction surface located in the separated flow gradually enhances. The power spectral, coherence, and phase analyses of the schlieren images describe the dominant oscillation structure and its relationship with other regions. During the separated flow, the pressure change in the subsonic separated region first lead to a change in the state of the separated shear layer, after which the shock waves in the shock train, move. The oscillatory flow is a process wherein the upstream shock wave oscillates, causing the entire downstream channel to fluctuate.

## Introduction

Research focusing on the development of modern aero-engines has mainly aimed at increasing the thrust-to-weight ratio to achieve high-speed flight and transportation of greater payloads^1–4^. Therefore, an important research direction is to increase the stage load of the compressor to reduce the number of compression stages required, enhance the single-stage pressure ratio, and improve the thrust-to-weight ratio of the engine^5^. By organizing the shock waves^6–9^ that occur in the channel for reasonable pressurization, a supersonic cascade^10^ that can obtain a high single-stage pressure ratio can be achieved, meeting the requirement of a high thrust-to-weight ratio in modern aero-engines.

Background wave structures^11,12^ that are generated by the cascade geometry exist in the channel when the downstream backpressure is extremely weak. The boundary layer that develops from the freestream flow remains attached during the downstream development process. The flow field state at this stage is known as steady flow^13^, unthrottled flow^14^ or attached flow^15^. As the backpressure increases, the impingement of the shock train^16^, which consists of a series of normal or oblique shocks on the boundary layer, causes the flow to separate. Shock train movement is typically accompanied by oscillations, such as self-excited oscillation^17^ and forced oscillation^18^, which are induced by the unsteady combustion downstream. The unsteady movement of the shock train is of practical importance because it may feed instabilities into the combustor and induce pressure fluctuations that generate noise and lead to fluctuations in the wall loads, both of which need to be minimized. The flow field state at this stage is called the shock train flow^13^, throttled flow^14^ or separated flow^18^. When the backpressure exceeds a critical value, the resulting overflow leads to changes in the internal flow, altering the flow capturing characteristics of the cascade and transforming the flow field state to unstarted flow^13^ or oscillatory flow ^14^, during which the relatively stable background waves and shock train are replaced by large-amplitude shock oscillations. The propagation of acoustic waves, shock movement, spillage, separation bubble deformation, and other factors in the closed feedback loop of oscillation^19^ implies that the oscillation mechanism is completely different from that of the shock train oscillation occurring at mild backpressure^20,21^. The accompanying unsteady aerodynamic and thermal loads can inevitably exert unfavorable effects on engine performance, and can even affect the structural safety and flight control of the aircraft in extreme cases. Therefore, it is particularly important that the flow field state is effectively predicted and detected if an engine is to be safely monitored and controlled.

Several researchers have focused on detecting the location of the shock train leading shock (STLS) to monitor the flow field state at mild backpressure. According to the flow mechanism, when the backpressure or inflow conditions vary and break the balance between the shock train, inflow condition, and backpressure, the location of the shock train changes to obtain a new balance. When the shock train moves upstream, the downstream flow is compressed by both the STLS and a series of subsequent reflected shock waves. A disturbed flow field exhibits completely different characteristics from those of an undisturbed flow field. A variety of methods for detecting the STLS location have been developed^22^. In general, there are six methods that can be used to estimate the STLS location: the pressure ratio method^23^, pressure increase method^24^, standard deviation method^25^, power spectral density method^26^, static pressure summation method^23^, and backpressure model method^23^. Kong et al.^27^ and Li et al.^28^ proposed a new method by which the STLS could be located using deep learning neural networks with discrete pressure measurements as inputs to obtain a reconstruction of the internal flow field. Image processing was then used to obtain the STLS location based on the reconstructed flow field. This method provides high-density information, improving the detection accuracy. To conduct in-depth research on the oscillation mechanism of a shock train, several researchers have focused on detecting the STLS location based on high-speed schlieren image processing. Li et al.^29^ obtained the real-time location of the STLS by performing binary, subtraction, and open processing on a series of schlieren images. The experimental location of the STLS obtained by Li et al.^29^ was then compared with the results predicted using a low-order dynamic model, which was a one-dimensional analysis approach based on the free interaction theory that could qualitatively analyze the shock train behavior. Xu et al.^30^ also captured the real-time location of the STLS using unsteady two-dimensional compressible Reynolds-averaged Navier–Stokes (RANS) simulations to study the motion characteristics of a shock train caused by linearly increasing backpressure. A sudden sharp forward movement of the shock train was observed when the separation point surmounted the reflection points in the background waves, implying that the forward motion characteristics of the shock train were related to the background wave structures. To obtain information regarding the frequency and correlations in the spatial evolution of the flow field, some researchers have extended their perspective to the time–frequency and correlation analyses of high-precision numerical simulations and high-resolution schlieren images. Agostini et al.^31^ computed the cross correlations occurring between the pressure fluctuations over the entire flow field and the streamwise location of the shock at the selected elevation in large-eddy simulations of the shock/boundary-layer interaction. The main space–time properties of the leading shock motions were described together with their links with other regions in the flow. The shock dynamics appeared to reflect the physical phenomena in all frequency ranges of the separation zone. Sartor et al.^32^ conducted Fourier analysis of a series of schlieren snapshots to precisely characterize the structure of the perturbations at low- and medium-frequencies. The low-frequency perturbations affected the entire shock wave, including the top of the recirculation bubble, with medium-frequency perturbations located mainly in the mixing layer. Through the power spectra, coherence, and phase analyses of high-frequency pressure signals and schlieren images, Wang et al.^33^ provided a local and global description of the low-frequency unsteadiness of the shock oscillation in an isolator with an inflow Mach number of 2.94. In this study, time–frequency analysis is applied to pressure signals and schlieren images to study the oscillation structures and their links with other regions in the cascade channel under different states of flow.

Previous studies^14^ used traditional analysis method of flow field, which matched the pressure and schlieren images one to one to reveal the flow mechanism. However, we found that the artificial selection of representative pressure–time series and schlieren images to analyze the flow mechanism may lead to the real situation being misled by the subjective selection process. In this study, time–frequency analysis is performed on a series of pressure–time series and schlieren images to avoid the process of subjective selection. Time–frequency analysis is first carried out on the pressure–time series at the local pressure transducer to reveal the frequency-amplitude information of the local flow field. Then, in order to reveal the evolution behavior of the entire flow field, time–frequency analysis is carried out on the intensity-time series on the schlieren image, including power spectra analysis, coherence analysis and phase analysis, so as to reveal the dominant spatial oscillation structure and the movement relationship between different spatial structures. Finally, after clarifying the time–frequency information of the flow field evolution, the typical pressure–time series is extracted to correspond with the schlieren images to further reveal the flow physics.

The rest of this paper is organized as follows: “Experimental conditions and measurements” briefly describes the supersonic cascade installed in a free-jet wind tunnel with high-frequency response pressure measurement and high-speed schlieren visualization. In “General description of the investigated cases”, the backpressure–time histories and the corresponding flow field structure of the studied case are introduced. In “Wall static pressure measurements”, the time evolution of the pressure fluctuation during the throttling process is analyzed based on the joint time–frequency results for pressure, and the spatial distributions of the pressure fluctuations in different flow field states are presented based on the results obtained by linear interpolation of the pressure power spectra at different positions. In “High-speed schlieren visualization”, the power spectrum, coherence, and phase analyses of the light intensity value–time series of all the pixels in the cascade channel are conducted to determine the oscillation structures and their links with other regions in the flow field under different flow field states. The flow mechanism is discussed in “Discussion on flow mechanism”. The conclusions are summarized in “Conclusions”.

## Experimental method

### Experimental conditions and measurements

**Figure 1:**
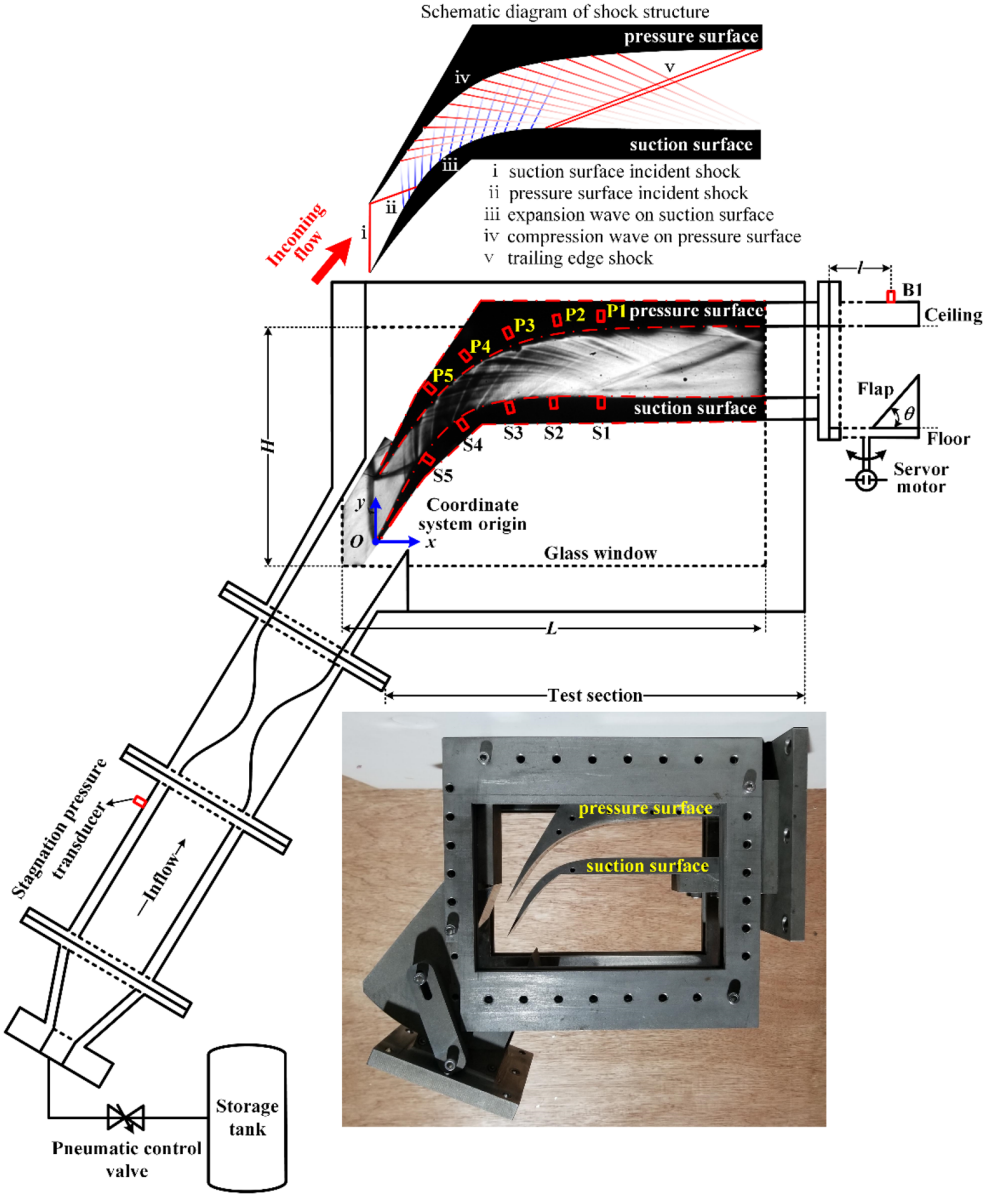
Schematic of the supersonic cascade installed in a free-jet wind tunnel with an instantaneous pressure measurement setup, experimental optical access and schematic diagram of shock structure^38^.

The SAV21 supersonic cascade model were tested in the free-jet-type wind tunnel at the Harbin Institute of Technology, as shown in Fig. 1.

The cascade was designed by the German Aerospace Center (DLR) with designed inflow Mach number of 2.4. Under the design condition, the shock waves in one cascade channel have little effect on the flow field in other cascade channels. Therefore, the single-channel experimental model of the SAV21 supersonic cascade was designed.

The upstream wind tunnel was comprised of a storage tank with a volume of 10 m^3^ and a pressure of ~ 13 Mpa, as shown in Fig. 1. The main geometrical parameters and freestream conditions of the wind tunnel are listed in Table 1.

**Table 1:** Main geometrical parameters and freestream conditions^38^.

Parameter	Value
Schlieren visual glass window height, *H* (mm)	120
Schlieren visual glass window length, *L* (mm)	180
Cascade width, *W* (mm)	40
Distance between backpressure transducer B1 and test section, *l* (mm)	140
Coordinate system origin, *O*	Tip of suction surface
*x*-Coordinates of S1, S2, S3, S4, and S5 (mm)	91.68, 71.68, 51.84, 33.49, 19.71
*x*-Coordinates of P1, P2, P3, P4, and P5 (mm)	91.68, 73.36, 53.95, 36.07, 21.62
Inflow Mach number, *M*_∞_	2.4 ± 0.5%
Inflow stagnation pressure, *P*_t_ (pa)	438,000 ± 0.5%
Inflow static pressure, *p*_∞_ (pa)	29,966 ± 0.5%
Inflow stagnation temperature, *T*_t_ (K)	287 ± 5
Inflow static temperature, *T*_∞_ (K)	133 ± 5
Unit Reynolds number, *Re* (/m)	4.8 × 10^7^

**Figure 2:**
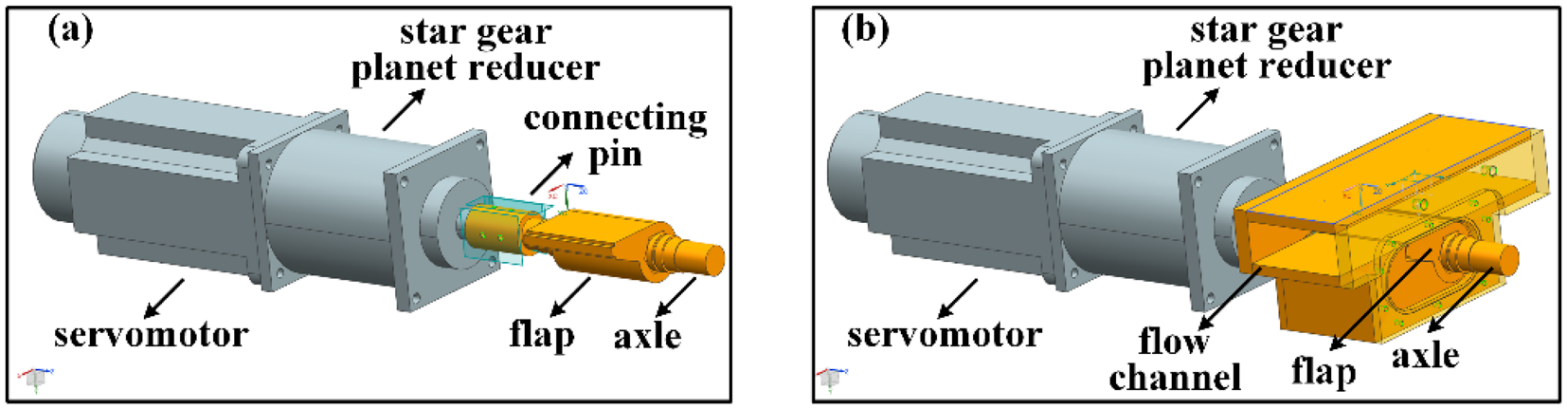
(**a**) The flap with its driven servomotor and (**b**) the flap installed in the flow channel.

In this study, a mechanical flap driven by a stepper motor was used for cold flow analysis^34–36^ to focus on the key impact factors of the flow mechanism. The flap was hinged to the wind tunnel ceiling at a location of *y* = 50 mm. To form a geometrical throat that chokes the flow to generate backpressure downstream, the increase of flap angle was driven using a servomotor (torque = 15 Nm, power = 2.3 kW) with a star gear planet reducer (reduction ratio = 6). The resolution of the flap angle was 0.006°. As shown in Fig. 2, when the servomotor is commanded to rotate, the servomotor rotates through a star gear planet reducer with a reduction ratio of 6 to drive the flap to rotate. Each unit of instruction transmitted to the servomotor causes the flap to rotate by 0.006°. The detail angle at which the flap rotates during the experiment and the resulting change in backpressure are discussed in “General description of the investigated cases”. Far away from the cascade, the flap creates a uniform downstream pressure distribution at the cascade outlet after choking the flow, which has been verified by the previous numerical simulations^37^.

The wall pressure measurements were made with ten fast-response transducers (effective frequency responses of approximately 20 kHz and range of 0–100 kPa). The pressure (suction) surface transducers, P1–P5 (S1 to S5), were arranged along the centerline of the pressure (suction) surface, 20 mm away from the sidewall. Setting the tip of the suction surface as the origin of the coordinate system, the *x*-coordinates of the transducers on the pressure surface and suction surface are listed in Table 1. The comprehensive precision of the transducers was ± 0.1% at full scale. The signals were recorded with a sampling frequency of 10 kHz using an IOtech 6220 voltage measurement module from Measure Computing Corp. Based on the experimental stagnation sonic speed (343.7 m/s), it can be conservatively estimated that the actual cutoff frequency is beyond 830 Hz.

The terminology—pressure surface and suction surface need to be clarified. As the name implies, the pressure surface is the blade surface on which the fluid is being pressed. The blade surface on which the fluid directly impinges (continuously approaches) after entering the flow channel is the pressure surface. The suction surface is the blade surface on which the fluid is being expanded. The blade surface on which the fluid gradually leaves after entering the flow channel is the suction surface. As shown in the schematic diagram of shock structure in Fig. 1, the fluid entering the cascade channel directly impinges the upper blade surface and gradually leaves the lower blade surface. Therefore, the upper and lower blade surfaces correspond to pressure surface and suction surface respectively.

The schematic diagram of shock structure in the cascade passage is shown in Fig. 1. A series of background waves generated on the cascade geometry profile such as suction surface incident shock, pressure surface incident shock, expansion wave on suction surface, compression wave on pressure surface and trailing edge shock. Schlieren images were captured using a high-speed camera (Photron V12) at a frame rate of 1000 fps with an exposure time of 250 μs and a resolution of 800 × 533 pixels. Illumination was provided using a 150 W halogen lamp with a pinhole approximately 2 mm in diameter. A convex lens with a focal length of 65 mm was placed in front of the source to focus the light at the location of the pin hole. A pair of concave mirrors with a focal length of 2.5 m was used to generate the parallel beam of light passing through the test section and refocus it at the knife-edge located in front of the high-speed camera. A pair of plane mirrors was also placed on either side of the test section to bend the path of the light.

The high-speed schlieren system was used to probe the transient structure of the flow field during the experiments. A Z-type light path that was reflected sequentially by two concave mirrors and cut by a circular knife-edge was adopted to visualize the following expression of the density gradient:

1 $$\begin{array}{*{20}l} {f\left( {\rho ,x,y} \right) = \sqrt {\left( {\frac{{{\text{d}}\rho }}{{{\text{d}}x}}} \right)^{2} + \left( {\frac{{{\text{d}}\rho }}{{{\text{d}}y}}} \right)^{2} } .} \\ \end{array}$$

A 5 V light-emitting diode (LED) light was installed on the optical window to synchronize the schlieren images with the pressure data. When the mechanical flap downstream is instructed to move, one measurement channel of the IOtech 6220 receives a high voltage signal at the same time and the LED light is turned on; this is captured by the high-speed camera. On the contrary, when the flap is ordered to stop, the voltage of the corresponding measurement channel of the IOtech 6220 decreases to 0, allowing the camera to capture the extinction of the LED light.

### General description of the investigated cases

**Figure 3:**
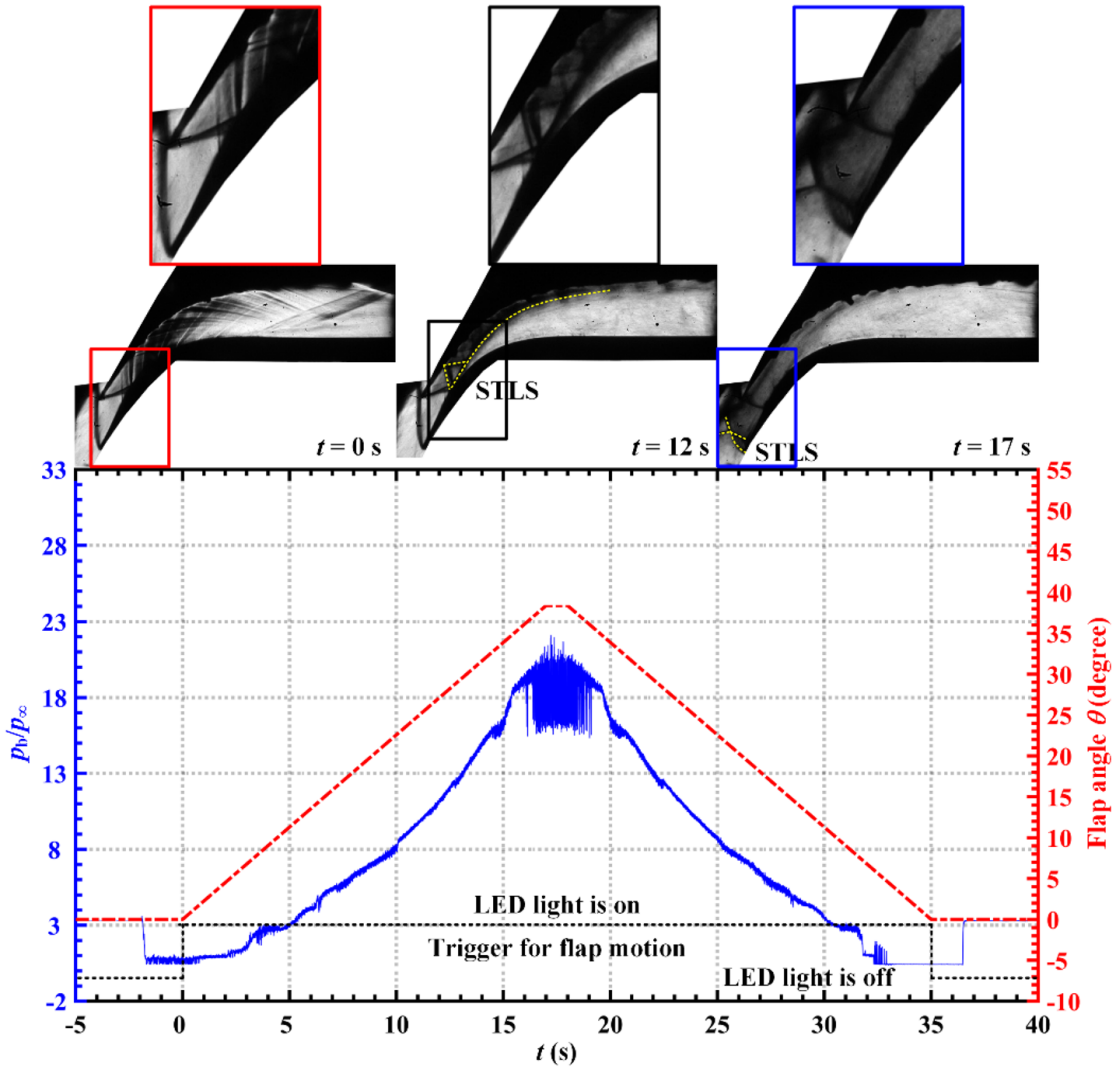
Normalized backpressure and the corresponding flap angle histories. Schlieren images exhibit the shock structure of the flow field at the corresponding time.

The pressure measured by the backpressure transducer (B1), or the backpressure (*p*_b_), is normalized by the inflow static pressure (*p*_∞_). The time histories of the normalized backpressure (*p*_b_/*p*_∞_) and the corresponding flap angle (*θ*) are depicted in Fig. 3.

When *t* < 0 s, the flap angle was maintained at 0°. When *t* = 0 s, the flap began to rotate at a speed of 2.259°/s for 17 s (the LED light was turned on simultaneously). The flap angle reached 38.4° at *t* = 17 s, and was maintained at 38.4° for 1 s. The flap angle began to drop at a speed of 2.259°/s at *t* = 18 s; this movement was maintained for 17 s. When *t* = 35 s, the flap angle was reduced to 0°, and the LED light turned off, marking the end of the throttling process. Thus, the experiment demonstrates the entire throttling process of attached flow–separated flow–oscillatory flow–separated flow–attached flow of the supersonic cascade.

## Result and discussions

### Wall static pressure measurements

#### Mean pressure profiles

**Figure 4:**
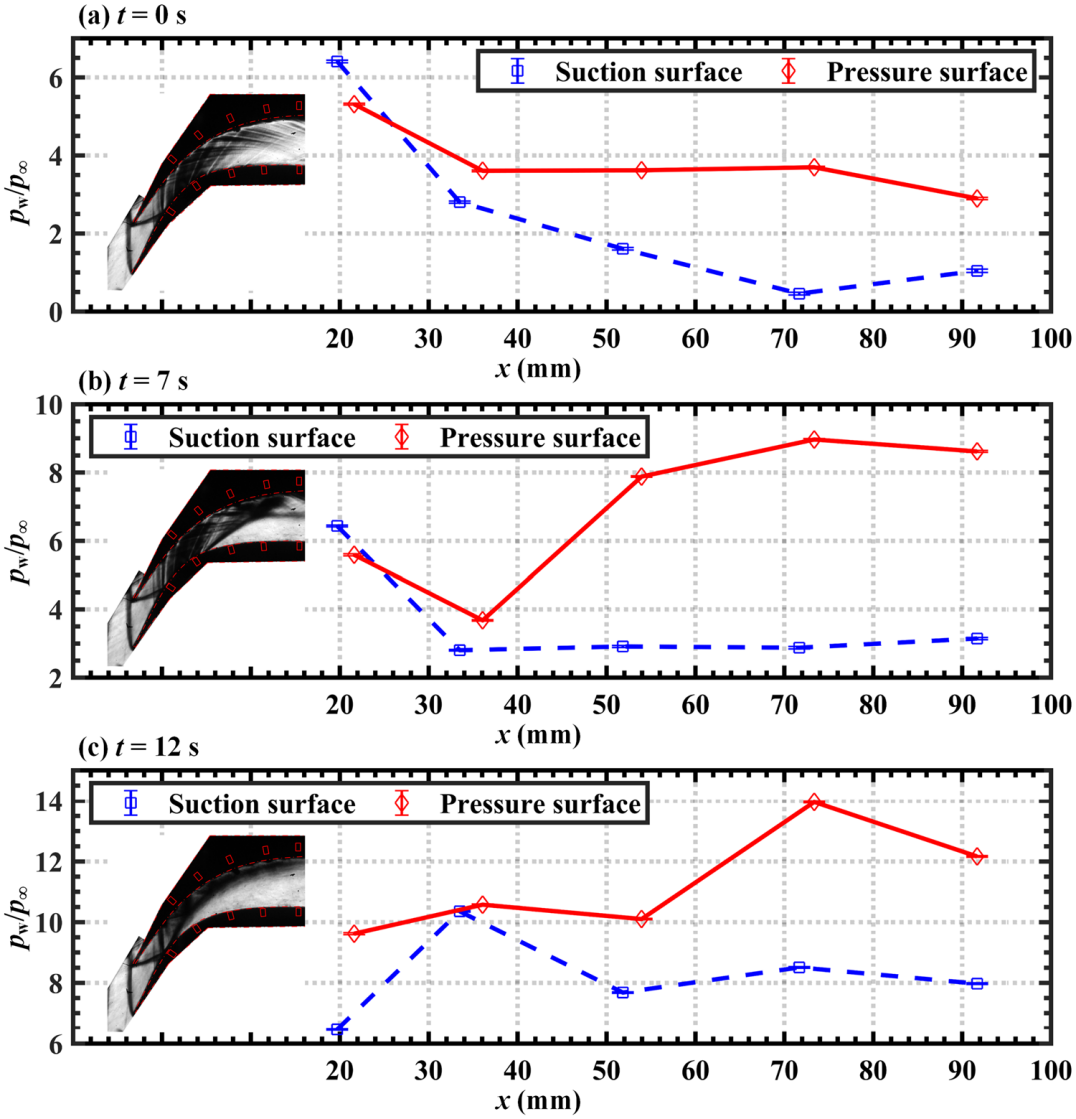
Normalized time-averaged wall pressure distributions along the center line of the supersonic cascade at (**a**) *t* = 0 s, (**b**) *t* = 7 s, and (**c**) *t* = 12 s. The schlieren images of the flow field at the corresponding times are presented on the left.

As depicted in Fig. 4, the normalized time-averaged wall pressure distribution (*p*_w_/*p*_∞_) is obtained by averaging 5000 wall pressure data points (*p*_w_) with a similar flow field structure and normalizing the distribution with the inflow static pressure (*p*_∞_). By using the least-squares linear fitting method and a high-precision pressure gauge, the transducers are calibrated according to their mean values before each run. The maximum deviation in the mean pressure is within 1%. Three measurements were taken under the same flow conditions to verify the repeatability of the pressure measurements. The root-mean-square deviation is calculated and error bars are added to indicate the (two-sided) 95% confidence interval, as depicted in Fig. 4.

Although it is simple and convenient to extract the pressure and schlieren image at an instantaneous moment, it is not representative due to the influence of random factors in the flow field. In this study, the pressure–time series of the quasi-steady flow field during the time interval of 0.5 s are averaged to accurately characterize the state of the flow field in this time interval. As illustrated in Fig. 4a, when *t* = 0 s, the flow field is unthrottled and the flap is at a downstream angle of 0°. A series of shock waves generated by the geometry of the cascade, called background waves^11^, are present within the channel at this time. The fluid passing through the cascade channel then undergoes an accelerated depressurization process. As depicted in Fig. 4b, the normalized backpressure (*p*_b_/*p*_∞_) increases to 5.38 at *t* = 7 s as the corresponding angle of the downstream flap reaches 15.813°. To match the high backpressure downstream, a shock train structure forms in the cascade channel. The impingement of the STLS on the suction surface causes a large area of separation and the supersonic core flow is deflected to the side of the pressure surface. Therefore, the pressure in the area downstream of the STLS is observed to increase significantly. As depicted in Fig. 4c, the normalized backpressure increases to 11.16, and the corresponding angle of the downstream flap reaches 27.108° at *t* = 12 s. The STLS then moves further upstream, resulting in the expansion of the subsonic separated flow and the contraction of the supersonic shock train flow.

#### Wall static pressure time series

**Figure 5:**
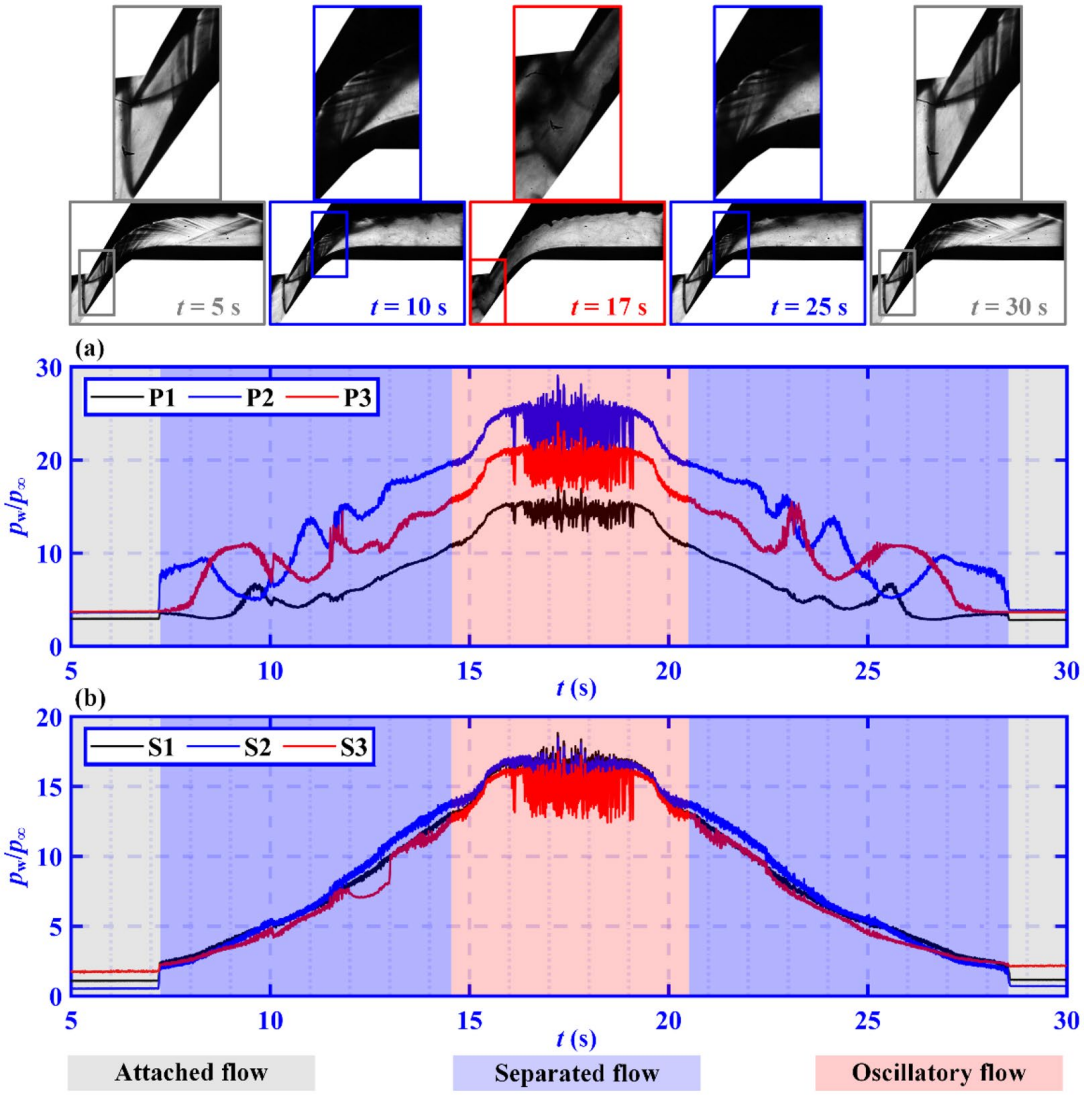
Typical normalized wall pressure–time histories for (**a**) the pressure surface and (**b**) the suction surface. The schlieren images of the flow field at the corresponding times are presented at the top.

Typical normalized wall pressure–time histories of the entire throttling process are depicted in Fig. 5. Throttling can be divided into five time periods and three flow stages based on the flow field structure and the pressure fluctuations.

The first time period of 0 s < *t* < 7.189 s is the stage in which the flow is attached, with the flow field characterized by the attachment of the boundary layer to both the pressure and suction surfaces. The second time period of 7.189 s < *t* < 14.602 s is the stage during which separated flow occurs, with a flow field characterized by a large region of subsonic separation near the suction surface. The pressure on the pressure surface located in the supersonic shock train flow fluctuates as a result of the sweep of the shock train, as indicated by the blue region in Fig. 5a. The pressure on the suction surface that is located in the subsonic separated flow increases almost linearly, as indicated by the blue region in Fig. 5b. The third period represents oscillatory flow, which begins when the STLS detaches from the tip of the pressure surface (*t* = 14.602 s), and ends when the STLS is re-attached to the tip of the pressure surface (*t* = 20.426 s). During the fourth time period (20.426 s < *t* < 28.522 s), the flow field recovers to become a separated flow without the occurrence of overflow. The fifth period begins when the boundary layer becomes attached to the suction surface (28.522 s < *t* ≤ 30.000 s), representing an attached flow.

#### Wall static pressure power spectra

A method combining the time and frequency analyses is required to analyze the evolution of the spectral energy in the pressure fluctuation over time. The continuous wavelet transform has proven to be efficient in investigating the buzz phenomenon that occurs in the supersonic inlet^22^. The synchrosqueezed transform used in this study can reduce energy smearing and enhance resolution in comparison with the continuous wavelet transform. During each of the analytical processes, the running average is first subtracted from the original pressure signal to obtain a zero-mean signal. In this study, a highpass filter with a passband frequency of 2 Hz is used for smoothing, such that other factors can be excluded and the features of the pressure fluctuation can be the focus. The synchrosqueezed transform of the zero-mean signal is computed using an analytic Morlet wavelet with a wave number of 18.

**Figure 6:**
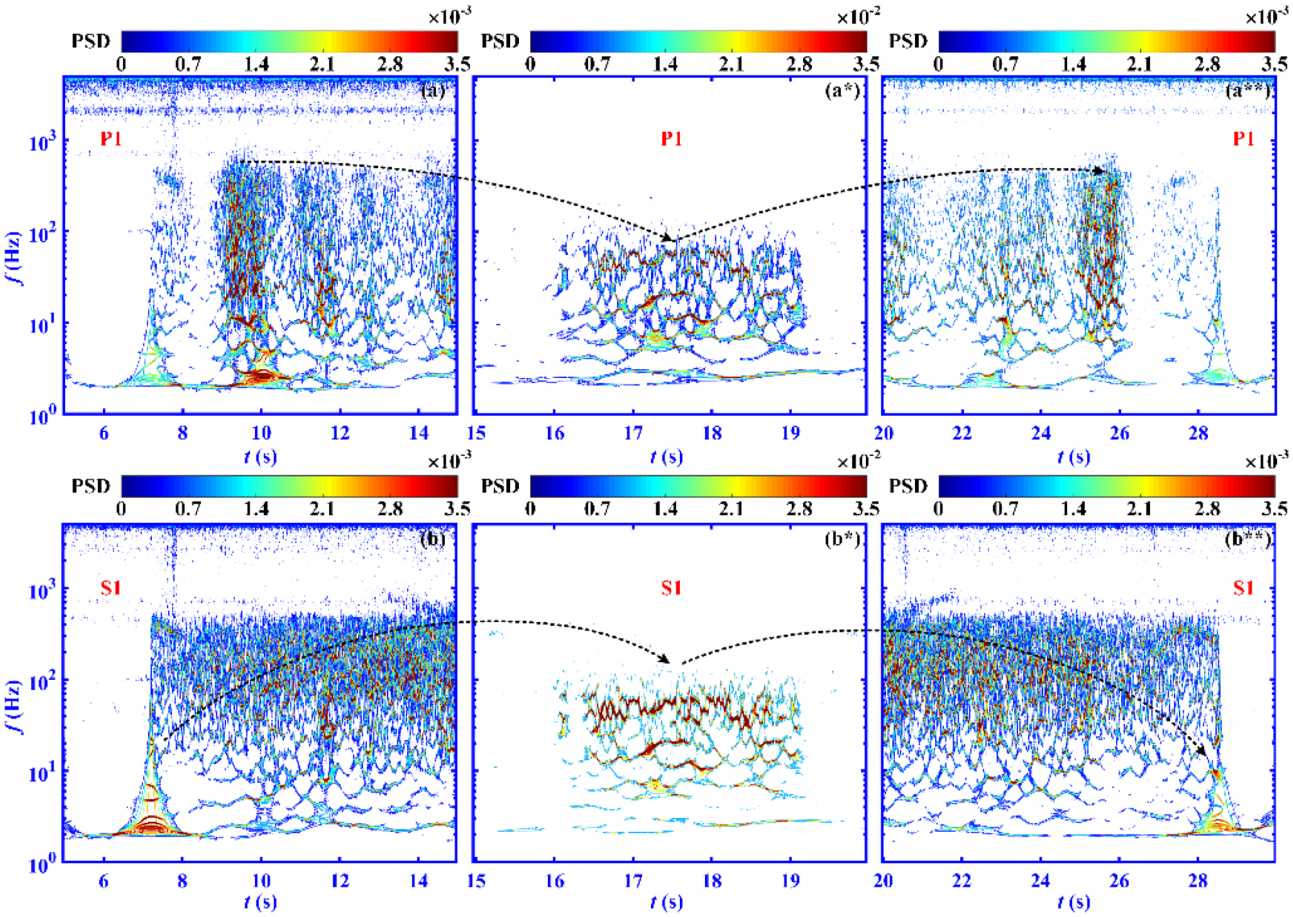
Power spectrum density contours of the pressure signals at P1 and S1 obtained using joint time–frequency analysis over the entire throttling process. Attached and separated flows observed in the pressure signals at (**a**) P1 and (**b**) S1 during the period 5 s < *t* < 15 s. Separated and oscillatory flows observed in the pressure signals at (a*) P1 and (b*) S1 during the period 15 s < *t* < 20 s. Separated and attached flows observed in the pressure signals at (a**) P1 and (b**) S1 during the period 20 s < *t* < 30 s.

The obtained time–frequency characteristics are represented as the power spectrum density (PSD) in Fig. 6. The points in the figure represent the energy of pressure fluctuation with a certain frequency at a certain moment. The closer the position downstream, the earlier the shock train induced by the downstream backpressure arrives. The pressure signals at the transducers that are furthest downstream, P1 and S1, are computed to demonstrate the time–frequency features of the throttling process. For clarity, the legends of the PSD contours containing the oscillatory flow (Fig. 6a*,b*) are ten times greater than those in Fig. 6a,b,a**,b**. The pressure fluctuation of P1 exhibits greater energy in the frequency range between 2 and 500 Hz during 9.04 s < *t* < 10.15 s of linear increase of backpressure (Fig. 6a) and 24.84 s < *t* < 26.11 s of linear decrease of backpressure (Fig. 6a**). This is caused by the sweep of the STLS moving upstream and downstream through P1. The energy involved in the pressure fluctuation at S1 gradually increases in the frequency range of 40–400 Hz during the linear increase in backpressure (Fig. 6b), and gradually weakens during the linear decrease in backpressure (Fig. 6b**). The shock train oscillation causes the volume of the subsonic separated flow, which has a finite mass, to change, which in turn induces the pressure fluctuation at S1. The separated region causes a ‘buffering effect’ because the fluctuation energy of the pressure inside it changes gradually. When oscillatory flow occurs (16 s < *t* < 17 s), as depicted in Fig. 6a*,b*, the dominant frequency range of the pressure fluctuation at P1 and S1 suddenly drops to 30 Hz < *f* < 60 Hz. As the backpressure increases further (17 s < *t* < 18 s), pressure fluctuations with greater energy and lower frequencies appear. The PSD distributions at P1 and S1 are similar during oscillatory flow, indicating that the flow field in the cascade channel oscillates as a whole.

The dashed arrows in Fig. 6 represents the evolution of dominant frequency of pressure fluctuation. According to the dashed arrows in Fig. 6a,a*,a**, the dominant frequency of pressure fluctuation on the pressure surface first decreases and then increases. For the time evolution of pressure fluctuation at P1, during the separated flow, the energy of pressure fluctuation is concentrated in the low-frequency shock train oscillation from 2 to 500 Hz. When the oscillatory flow occurs, the dominant frequency of pressure fluctuation suddenly decreases to 30 Hz < *f* < 60 Hz. When the separated flow is restored, the dominant frequency of pressure fluctuation increases to 2 Hz < *f* < 500 Hz. According to the dashed arrows in Fig. 6b,b*,b**, the dominant frequency of pressure fluctuation on the suction surface experiences a process of increasing, decreasing, increasing and decreasing. The oscillation of STLS around S1 results in an increase in pressure fluctuation energy at S1 in the frequency range of 40 Hz < *f* < 400 Hz. When the STLS passes over S1, the dominant frequency of pressure fluctuation at S1 in the separated flow increases gradually. When the oscillatory flow occurs, the dominant frequency suddenly decreases to between 30 and 60 Hz. When the separated flow is restored, the dominant frequency rise to around 100 Hz. As the STLS approaches S1, the dominant frequency decreases gradually.

**Figure 7:**
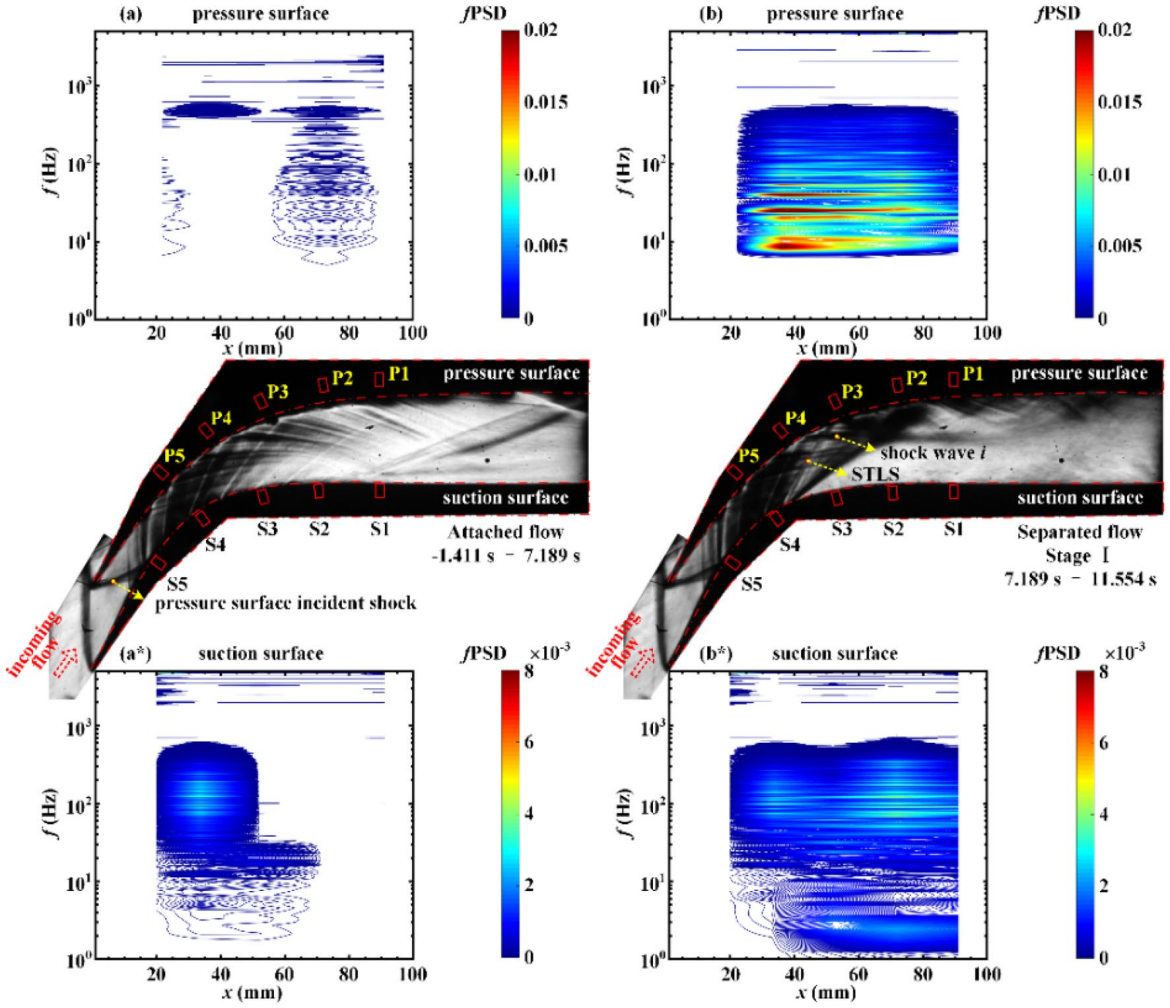
Contours of *f*PSD at the (**a**) pressure surface and (a*) suction surface in the attached flow during the period − 1.411 s < *t* < 7.189 s. Contours of *f*PSD at the (**b**) pressure surface and (b*) suction surface in the separated flow during the period 7.189 s < *t* < 11.554 s.

**Figure 8:**
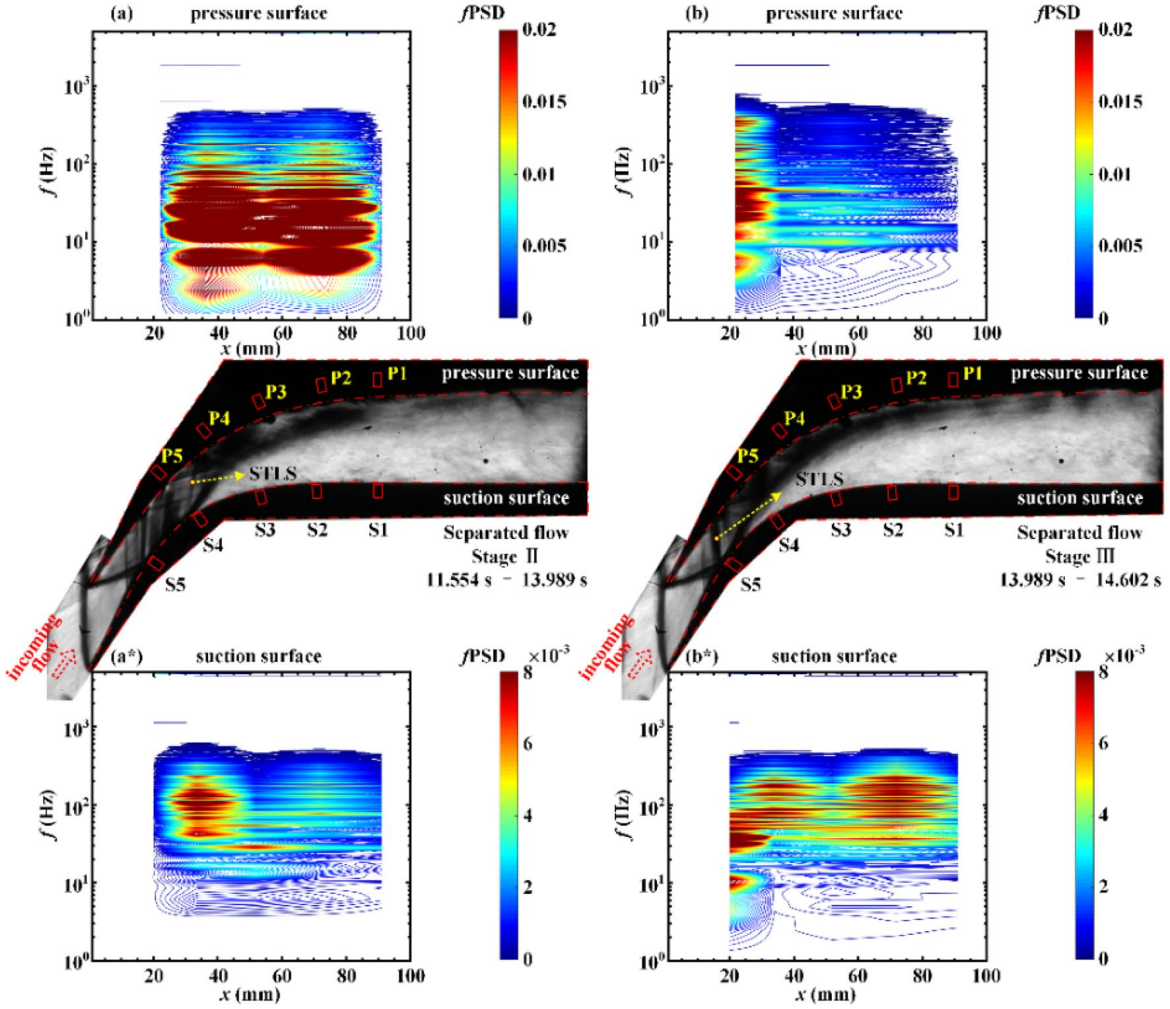
Contours of *f*PSD at the (**a**) pressure surface and (a*) suction surface in the separated flow during the period 11.554 s < *t* < 13.989 s. Contours of *f*PSD at the (**b**) pressure surface and (b*) suction surface in the separated flow during the period 13.989 s < *t* < 14.602 s.

**Figure 9:**
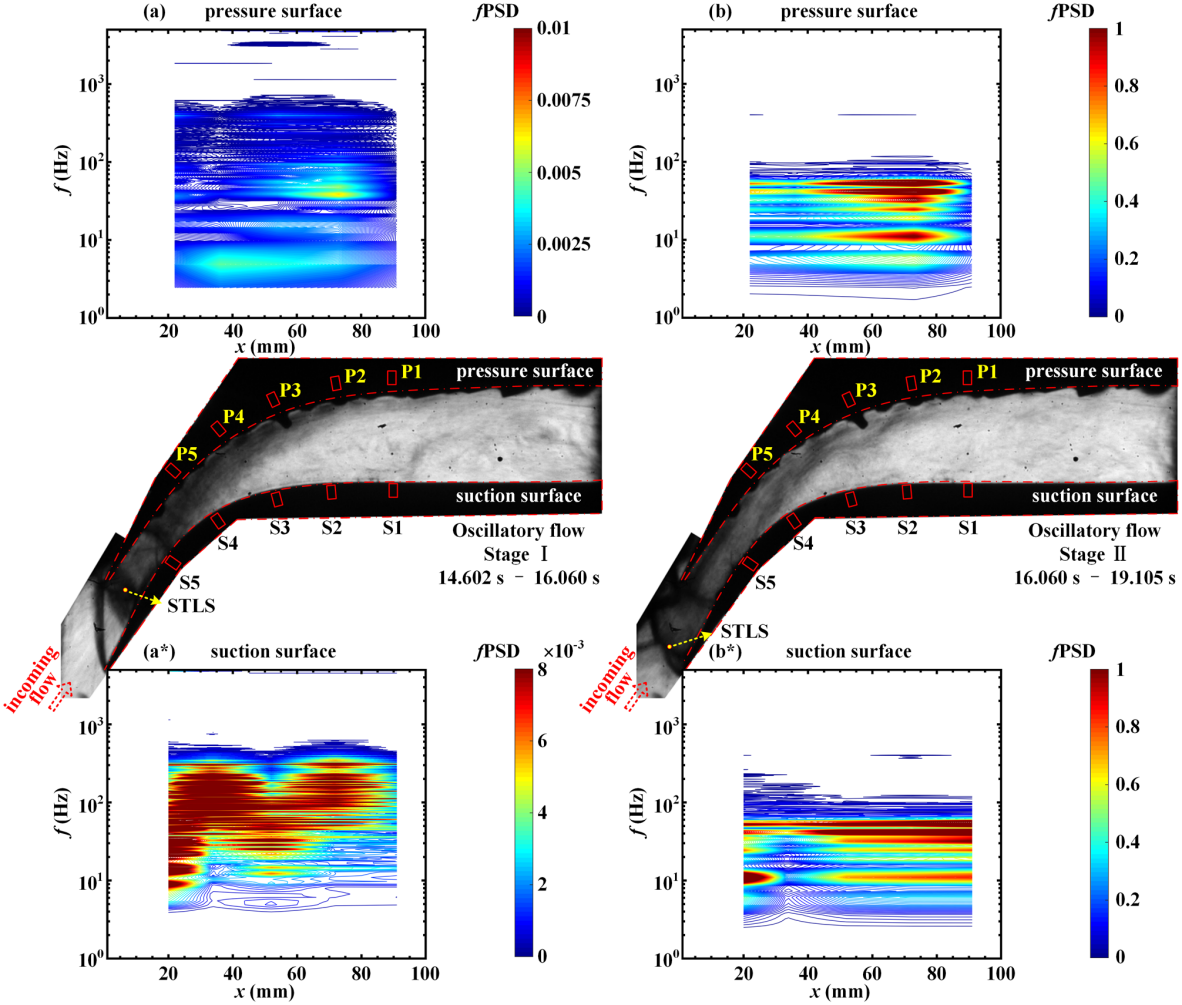
Contours of *f*PSD at the (**a**) pressure surface and (a*) suction surface in the oscillatory flow during the period 14.602 s < *t* < 16.060 s. Contours of *f*PSD at the (**b**) pressure surface and (b*) suction surface in the oscillatory flow during the period 16.060 s < *t* < 19.105 s.

The spatial distributions of the pressure fluctuation in the throttling process are depicted in Figs. 7, 8, and 9. After the zero-mean pressure signal *p*(*t*) is obtained, the Fourier transform of *p*(*t*) is computed, using a fast Fourier transform (FFT) algorithm with a Hamming window function with 50% overlap. The results of the Fourier analysis are ultimately represented as the weighted PSD (*f*PSD). This representation, called the premultiplied spectrum, provides access to the frequency content of the energy fluctuations. The values in the *f*PSD contours between the transducers are obtained by linear interpolation. The verification of interpolation methods is discussed in the “Supplementary Information”.

The separated flow is divided into three stages that depend on whether the STLS reaches S3, S4, or S5. The oscillatory flow is divided into two stages that are associated with the moment at which the STLS is detached from the pressure surface and suction surface. The results of the Fourier analysis for the attached flow (Fig. 7a,a*) indicate pressure fluctuation that is caused by impingement with the background waves. The pressure fluctuations exhibit energy content in the low-frequency shock oscillation frequency range of 4–550 Hz. The pressure fluctuations at S4 and S5 that are caused by the oscillation of the pressure surface incident shock demonstrate a fluctuation energy of (*f*PSD)_max_ = 0.0039 at the dominant frequency of 93.99 Hz, as depicted in Fig. 7a*. As illustrated in Fig. 7b, during stage I of the separated flow, the sweep of the shock train results in an increase in the pressure fluctuation energy on the pressure surface in the frequency range of 6–600 Hz. The oscillation of the upstream shock wave *i* results in P4 receiving the maximum fluctuation energy (*f*PSD)_max_ = 0.031 at the dominant frequency of 24.41 Hz. As depicted in Fig. 7b*, the pressure fluctuation energy in the downstream region of the STLS on the suction surface is enhanced, and the oscillation of the STLS causes S3 to receive the maximum fluctuation energy of *(f*PSD)_max_ = 0.0043 at the dominant frequency of 68.36 Hz.

During stage II of the separated flow in Fig. 8a, the oscillation of the shock train, which is located between P2 and P4, leads to a significant increase in the pressure fluctuation energy. The sweep of the STLS results in P4 receiving the maximum fluctuation energy (*f*PSD)_max_ = 0.15 at the dominant frequency of 28.08 Hz. As depicted in Figs. 8b and 9a, the backpressure increases further and the shock waves in the shock train move upstream of the transducers on the pressure surface. The shock train continues to move upstream and the pressure fluctuation energy at P1–P5 gradually weakens. There are some differences in the pressure fluctuations on the suction surface. With the increase in backpressure, the pressure fluctuation energy on the suction surface gradually increases in the frequency range of 4–600 Hz, as depicted in Figs. 8a*,b*, and 9a*. This is consistent with the results of the joint time–frequency analysis. The increase in backpressure leads to the intensification of shock train oscillation, which results in an increase in the volume-varying amplitude of the subsonic separation region and enhancement in the pressure fluctuation at S1–S5. When stage II of the oscillatory flow begins, large-amplitude pressure fluctuations occur in the frequency range below 60 Hz, as depicted in Fig. 9b,b*. The similarity in the spatial distribution of the pressure fluctuation spectral energy on the pressure surface and the suction surface indicates that the flow field oscillates as a whole.

The maximum fluctuation energy (*f*PSD)_max_ and the dominant frequency in each stage are summarized in Tables 2 and 3. Table 2 indicates that (*f*PSD)_max_ first increases and then decreases on the pressure surface, which represents the process in which the shock train first approaches the transducers on the pressure surface and then continues to move upstream. Table 3 indicates that (*f*PSD)_max_ continues to increase on the suction surface, which represents an ever-increasing oscillation in the shock train and the resulting increase in the volume-reliant amplitude of the subsonic separation region. A large-amplitude pressure fluctuation occurs at 52.49 Hz over the entire flow field during stage II of the oscillatory flow.

**Table 2:** Spectral evolution of pressure fluctuations on pressure surface during throttling process.

	(*f*PSD)_max_	Dominant frequency (Hz)	Location
Attached flow	0.0030	627.44	P2
Separated flow stage I	0.031	24.41	P4
Separated flow stage II	0.15	28.08	P4
Separated flow stage III	0.043	46.39	P5
Oscillatory flow stage I	0.013	39.06	P2
Oscillatory flow stage II	2.17	52.49	P2

**Table 3:** Spectral evolution of pressure fluctuations on suction surface during throttling process.

	(*f*PSD)_max_	Dominant frequency (Hz)	Location
Attached flow	0.0039	93.99	S4
Separated flow stage I	0.0043	68.36	S3
Separated flow stage II	0.017	125.73	S4
Separated flow stage III	0.023	39.06	S5
Oscillatory flow stage I	0.028	45.78	S5
Oscillatory flow stage II	1.13	52.49	S1

### High-speed schlieren visualization

**Figure 10:**
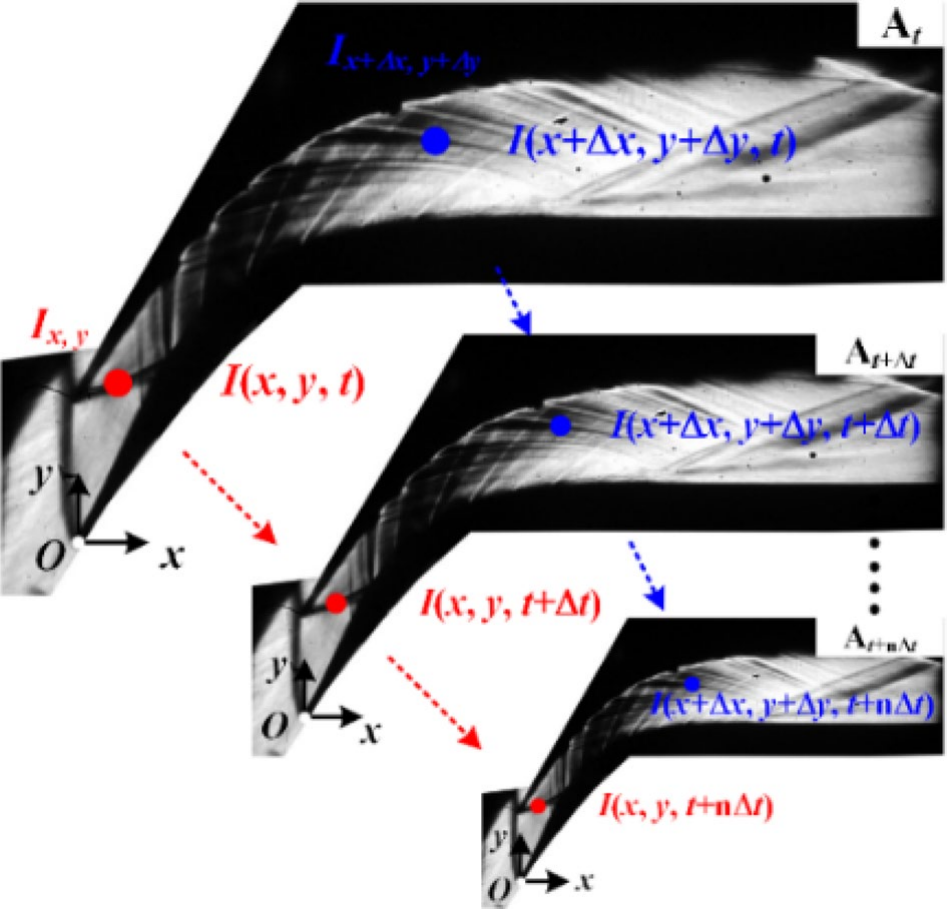
Method used for the time–frequency analysis of the schlieren images.

In this section, time–frequency analysis is performed on the schlieren images obtained by the high-frame-rate camera to characterize the structure of the oscillation and its relationship with the other regions. The method used in the time–frequency analysis of the schlieren images is depicted in Fig. 10. By comparing the distance of a pixel at a fixed point to the origin in the image with the actual distance, the relationship between the pixel coordinates (*i*, *j*) and the actual coordinates (*x*, *y*) can be obtained. Using the synchronization method described above, the sequence of images can be related to the actual time (*t*). In this study, the values of pixels *i* and *j* vary from 1 to 800 and 1–533, respectively, corresponding to the actual horizontal and vertical coordinates of *x* and *y* that range from − 15 to 165 mm and − 10 to 110 mm*,* respectively. At a given time, the light intensity value at a given position can be denoted as *I*(*x*, *y*, *t*). The time series that describes the evolution of the light intensity at a given position (*x*, *y*) can be denoted as *I*_*xy*_. The premultiplied spectrum, two-point coherence*,* and phase spectrum of *I*_*xy*_ are calculated in this section. The calculation results of *I*_*xy*_ at all positions correspond with the contours of *f*PSD, coherence coefficient, and phase values of the entire flow field at all frequencies. The contours of the flow field at the dominant frequency can be extracted to analyze the main oscillation structure and its spatial evolutionary relationship with the other regions.

#### Power spectra analysis

**Figure 11:**
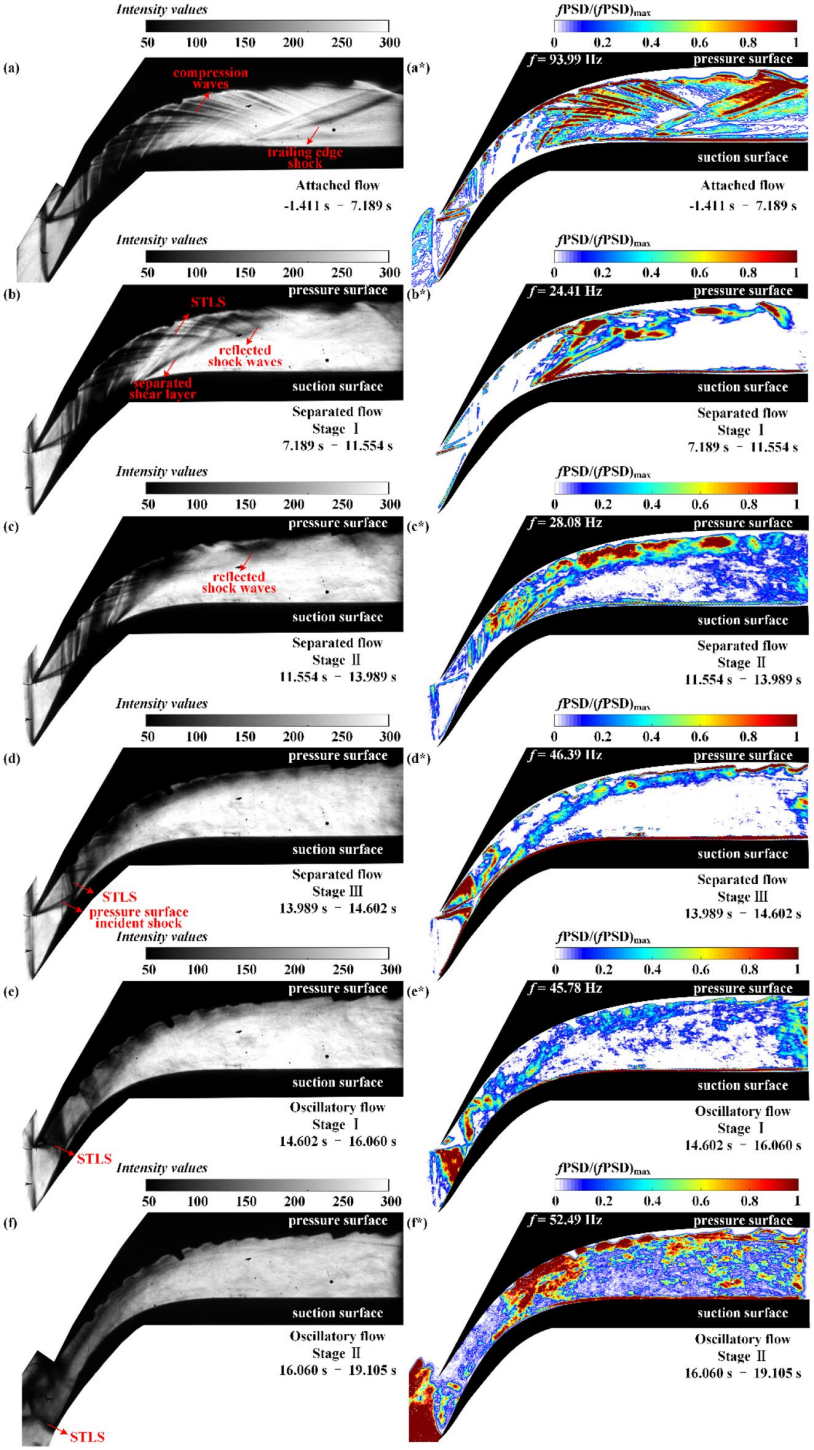
Schlieren images of the flow field during (**a**) attached flow, (**b**) stage I of separated flow, (**c**) stage II of separated flow, (**d**) stage III of separated flow, (**e**) stage I of oscillatory flow, and **(f)** stage II of oscillatory flow. The *f*PSD contours of flow field during (a*) attached flow, (b*) stage I of separated flow, (c*) stage II of separated flow, (d*) stage III of separated flow, (e*) stage I of oscillatory flow, and (f*) stage II of oscillatory flow at the corresponding dominant frequency.

The *f*PSD contours at the corresponding dominant frequency during the attached flow, separated flow and oscillatory flow are depicted in Fig. 11.

On the left side of the *f*PSD contours, the schlieren images at the same stage are drawn to clarify the oscillation structures. The main oscillation structures in the background wave during the attached flow are displayed in Fig. 11a*. The compression wave region and the trailing edge shock exhibit higher *f*PSD values, indicating that the oscillation of the compression waves and the trailing edge shock is violent. The main oscillation structure that occurs during separated flow is the shock train, including the STLS, separated shear layer, and reflected shock waves, as depicted in Fig. 11b,b* that demonstrate stage I of the separated flow. The oscillations in these three structures are affected by the background waves during the upstream movement of the shock train. Because the expansion waves generated on the suction surface weaken the oscillation of the STLS and the separated shear layer in stage II of the separated flow, the main oscillation structures are the reflected shock waves depicted in Fig. 11c,c*. Owing to the greater intensity of the STLS at this point, the reflected shock waves are weaker and may even disappear in stage III of the separated flow, leaving the STLS, separated shear layer, and pressure surface incident shock as the main oscillation structures, as depicted in Fig. 11d,d*. As illustrated in Fig. 11e,e*, the area of high *f*PSD near the tip of the pressure surface represents the oscillating region of the STLS in stage I of the oscillatory flow. As depicted in Fig. 11f,f*, the area of high *f*PSD near the suction surface tip indicates the large-amplitude oscillation of the STLS in stage II of the oscillatory flow*,* which causes fluctuation of the flow field in the downstream cascade channel.

#### Coherence and phase analysis

After understanding the structure of the main oscillation in the flow field, further coherence and phase analyses are conducted to determine the relationship between the evolution of the different oscillation structures at the dominant frequency. The coherence *C*_αβ_(*f*) between two time signals, α(*t*) and β(*t*), is defined as

2 $$C_{{\upalpha \upbeta }} (f) = \frac{{\left| {P_{{\upalpha \upbeta }} (f)} \right|^{2} }}{{P_{{\upalpha \upalpha }} (f)P_{{\upbeta \upbeta }} (f)}},$$

where *P*_αα_(*f*) and *P*_ββ_(*f*) denote the self-PSD of signals α(*t*) and β(*t*), respectively*,* and *P*_αβ_(*f*) denotes the cross-PSD between signals α(*t*) and β(*t*). *P*_αβ_(*f*) can be expressed using

3 $$P_{{\upalpha \upbeta }} (f) = G_{{\upalpha \upbeta }} (f) - jQ_{{\upalpha \upbeta }} (f) = \left| {P_{{\upalpha \upbeta }} (f)} \right|e^{{ - jQ_{{\upalpha \upbeta }} (f)}} ,$$

where *G*_αβ_(*f*) and *Q*_αβ_(*f*) are the real and imaginary parts of *P*_αβ_(*f*), respectively. Based on the coherence spectrum, further phase analysis can be used to obtain the lead–lag relationship between the fluctuations in the two time signals at the associated frequency, with the corresponding value in the form of angles or radians. The phase *θ*_αβ_(*f*) between two time signals, α(*t*) and β(*t*), is defined as

4 $$\theta _{{\upalpha \upbeta }} (f) = \arctan \frac{{Q_{{\upalpha \upbeta }} (f)}}{{G_{{\upalpha \upbeta }} (f)}}.$$

When oscillation occurs at a fixed frequency *f*, the phase *θ* in angular form caused by the time delay τ can be calculated by

5 $$\theta = 360f \uptau .$$

For all frequencies, the coherence satisfies

6 $$0 \le C_{{\upalpha \upbeta }} (f) \le 1.$$

**Figure 12:**
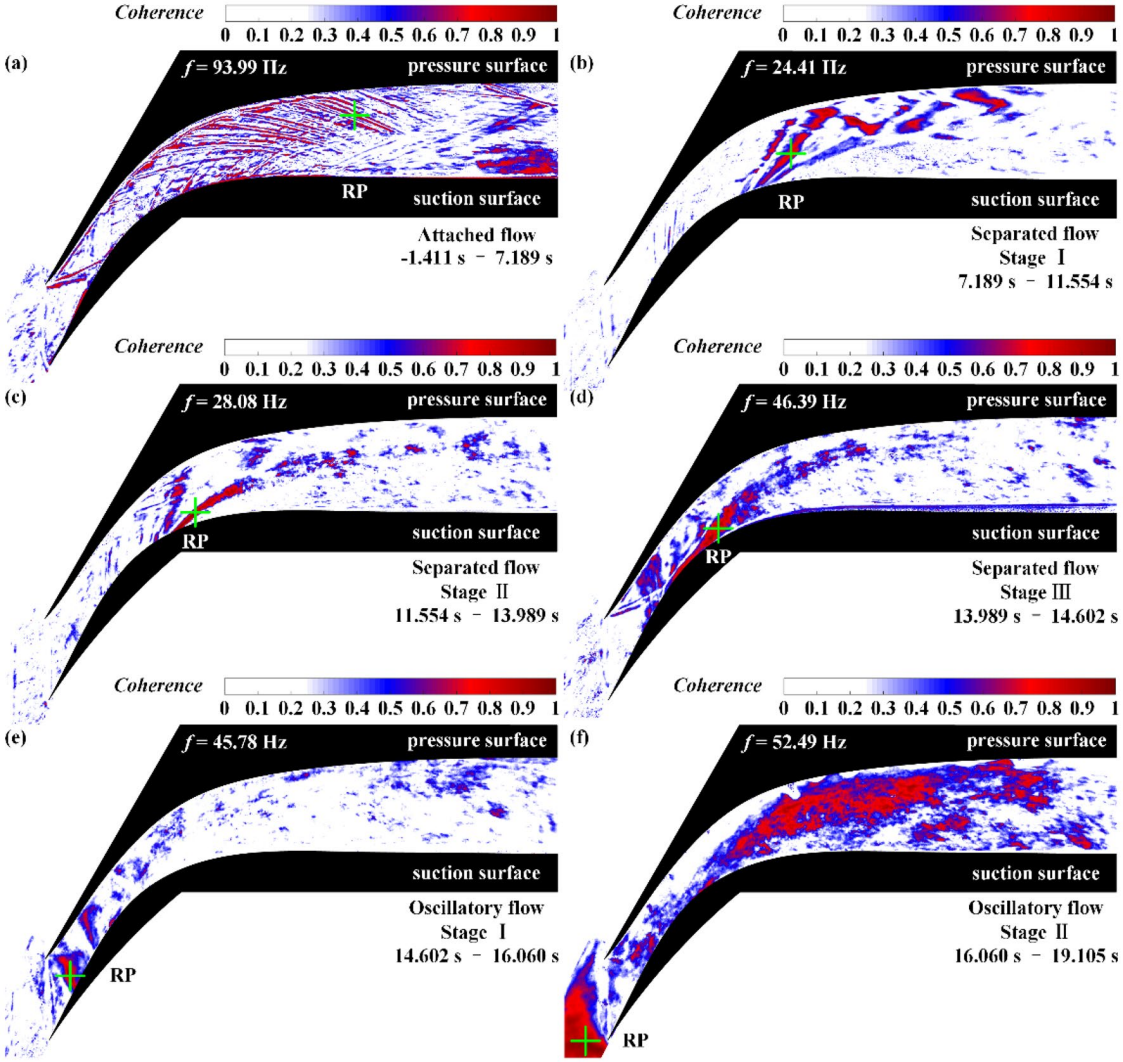
Coherence contours of the flow field during (**a**) attached flow, (**b**) stage I of separated flow, (**c**) stage II of separated flow, (**d**) stage III of separated flow, (**e**) stage I of oscillatory flow, and (**f**) stage II of oscillatory flow. The symbol + represents the reference point (RP).

**Figure 13:**
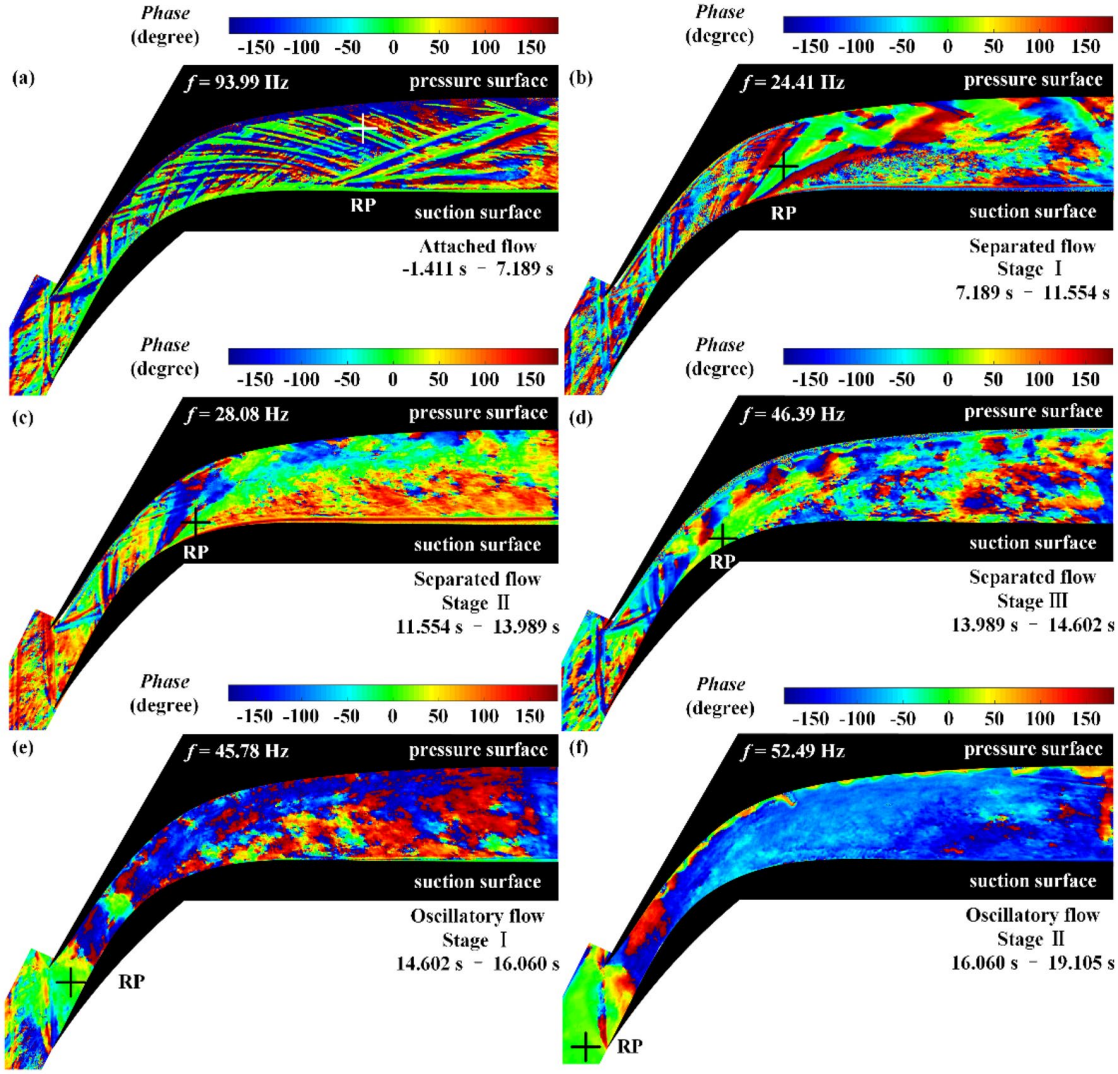
Phase contours of the flow field during (**a**) attached flow, (**b**) stage I of separated flow, (**c**) stage II of separated flow, (**d**) stage III of separated flow, (**e**) stage I of oscillatory flow, and (**f**) stage II of oscillatory flow. The symbol + represents the reference point (RP).

When *C*_αβ_(*f*) = 1, the time series α(*t*) and β(*t*) are linearly related at frequency *f* in the sense of the convolution filter; when *C*_αβ_(*f*) = 0, they are unrelated. In the coherence contours (Fig. 12), a large coherence value at a particular location indicates that the variation in the flow field at that location is highly coupled to that at the reference point. In the phase contours (Fig. 13), a large positive value for the phase at a particular location indicates that the event first occurs at that location relative to the reference point.

In analyzing the coherence contours of the separated flow, depicted in Fig. 12b–d, it is found that the motion of the STLS, separated shear layer, and reflected shock waves are highly coupled. Upon further observation of the phase contours in the separated flow, depicted in Fig. 13b–d, it is found that the phase of the separated shear layer is greater than that of the STLS and the reflected shock waves by more than 100°. The phase results indicate that the pressure change in the subsonic separation region near the suction surface first leads to a change in the state of the separated shear layer followed by changes in the states of the shock wave structures in the shock train, including both the STLS and the reflected shock waves.

In stage I of the oscillatory flow, as depicted in Fig. 12e, only a small region with high coherence can be observed near the tip of the pressure surface, while the large-amplitude shock oscillation results in highly coupled flow field fluctuations throughout the cascade channel during stage II of the oscillatory flow, as depicted in Fig. 12f. Further observation of the phase contours in Fig. 13e,f reveals that the phase of the upstream oscillation region of the STLS is higher than that of the flow field in the downstream cascade channel by 100°–150°. This indicates that the upstream oscillation of the STLS occurs first, leading to a large oscillation in the flow field in the downstream cascade channel.

### Discussion on flow mechanism

**Figure 14:**
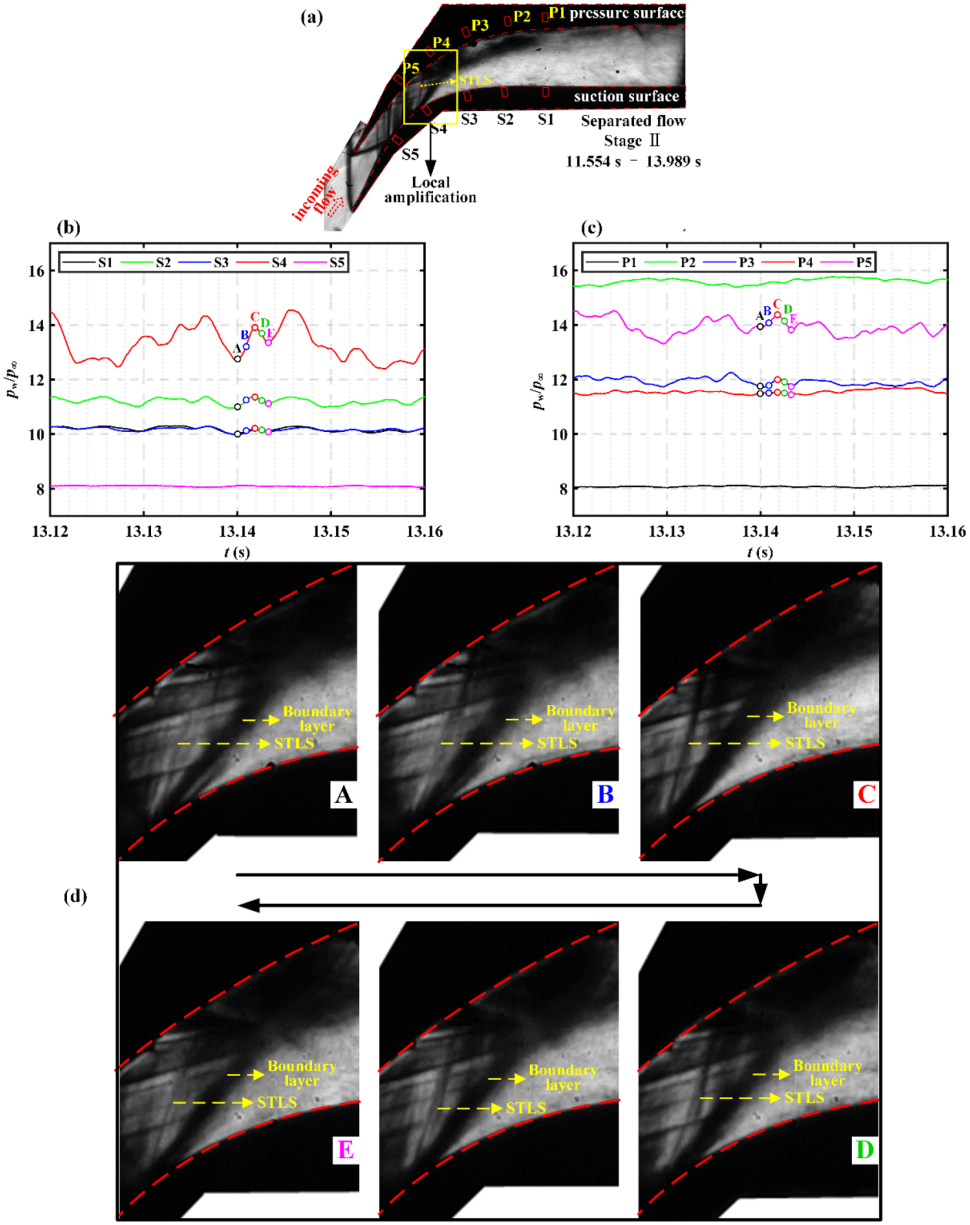
(**a**) Schlieren image of the entire flow field in the stage II of separated flow. Pressure–time series on the (**b**) suction surface and (**c**) pressure surface in the stage II of separated flow. (**d**) Schlieren images corresponding to the pressure–time series.

The conclusions obtained from time–frequency analysis based on pressure–time series and schlieren images in “Wall static pressure measurements” and “High-speed schlieren visualization” are further discussed in this section to reveal the flow physics. Figure 14 shows the evolution process of the flow field in the separated flow. Figure 14a shows the schlieren image of the entire flow field in the separated flow.

Based on the conclusions obtained in Fig. 11, the dominant oscillation structure is amplified in Fig. 14d to describe the flow field evolution within an oscillation period. When the flow field evolves from moment A to moment C, as shown in Figs. 13c and 14b, the pressure in the downstream flow field of STLS increases. As shown in Fig. 14d, during the evolution of the flow field from A to C, the color of STLS darkens and the shock angle increases, which indicates that the STLS is enhanced to generate a larger pressure rise. As shown in Fig. 14d, during the evolution of the flow field from C to E, the color of STLS becomes lighter and the shock angle decreases, which indicates the STLS is weakened. This is consistent with the decrease in pressure from moment C to moment E as shown in Fig. 14b,c. As shown in Fig. 14b,c, the closer to the STLS, the greater the pressure fluctuation is. This is because in the shock train, the strength of STLS is the largest and the pressure fluctuation caused by its oscillation is the strongest.

Based on the one-to-one correspondence between pressure and schlieren, the flow physics of the separated flow is revealed. The flow field evolution of the separated flow is dominated by the shock motion. Since the STLS has the greatest strength, it causes the largest pressure fluctuation and is also the dominant oscillation structure.

**Figure 15:**
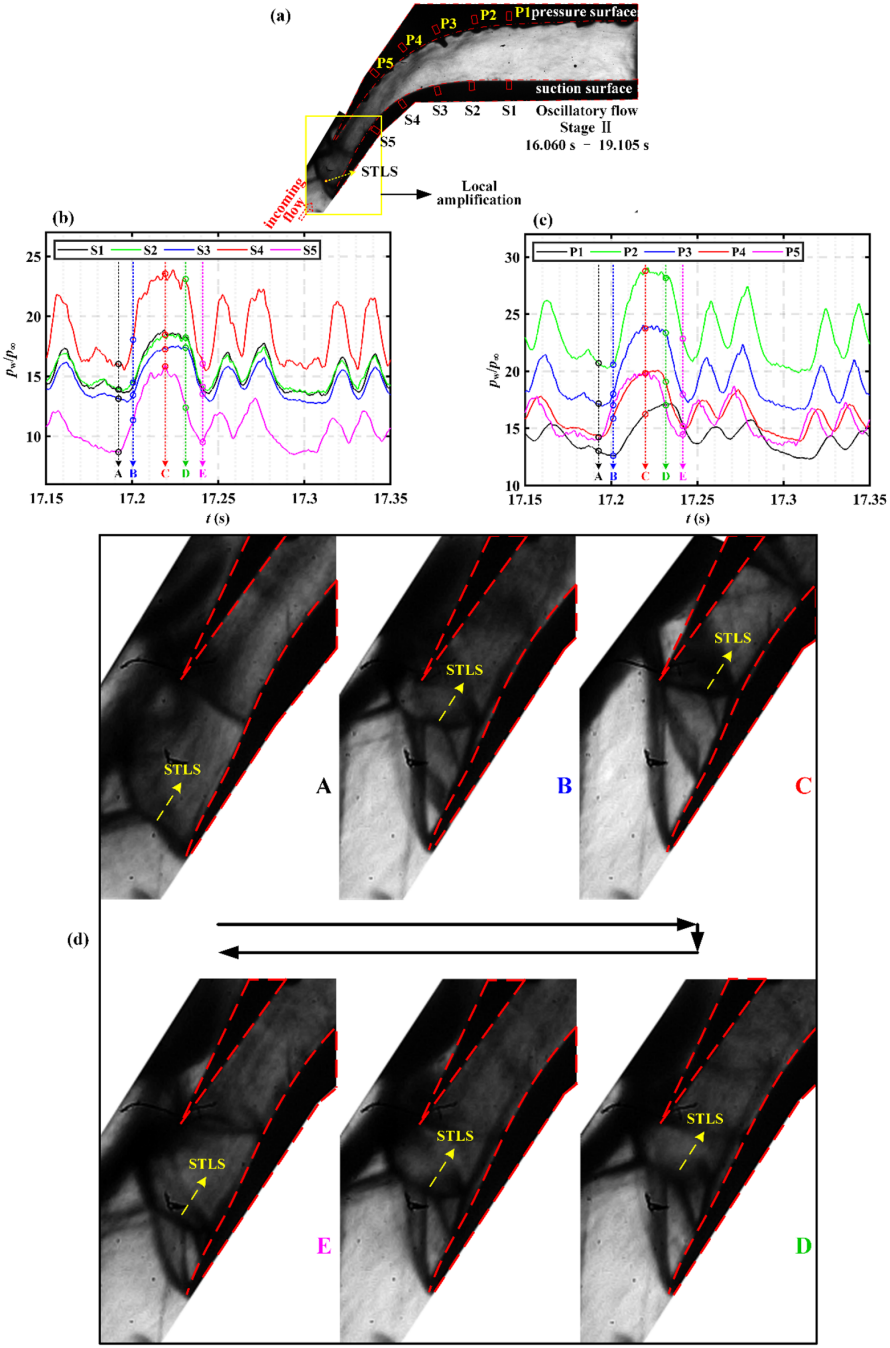
(**a**) Schlieren image of the entire flow field in the stage II of oscillatory flow. Pressure–time series on the (**b**) suction surface and (**c**) pressure surface in the stage II of oscillatory flow. (**d**) Schlieren images corresponding to the pressure–time series.

Figure 15a shows the schlieren image of the entire flow field in the oscillatory flow. Based on the conclusions obtained in Fig. 11, the dominant oscillation structure is amplified in Fig. 15d to describe the flow field evolution within an oscillation period. As shown in Fig. 15d, at moment A, the STLS is detached from the pressure surface tip and is located at the suction surface tip. The larger detachment distance leads to a larger amount of overflow, which in turns results in a lower pressure, as shown in Fig. 15b,c. When the flow field evolves from moment A to moment C, the STLS gradually approaches the pressure surface tip, leading to the decrease of the amount of overflow. This further leads to an increase in pressure, as shown in Fig. 15b,c. When the flow field evolves from moment C to moment E, as shown in Fig. 15d, the detachment distance of STLS to the pressure surface tip increases, which leads to the increase of the amount of overflow. This further results in a drop in pressure, as shown in Fig. 15b,c. As shown in Fig. 15b,c, the pressure fluctuation at the upstream S5 (P5) is earlier than that at the downstream. This is consistent with the conclusion obtained in Fig. 13. In the oscillatory flow, the upstream flow field evolves first, and then changes the downstream flow field.

Based on the one-to-one correspondence between pressure and schlieren, the flow physics of the oscillatory flow is revealed. In the oscillatory flow, the feedback mechanism of the flow field evolution mainly includes the shock motion and overflow. These upstream flow field variations propagate to the downstream, resulting in subsequent fluctuations of the entire downstream flow field.

**Figure 16:**
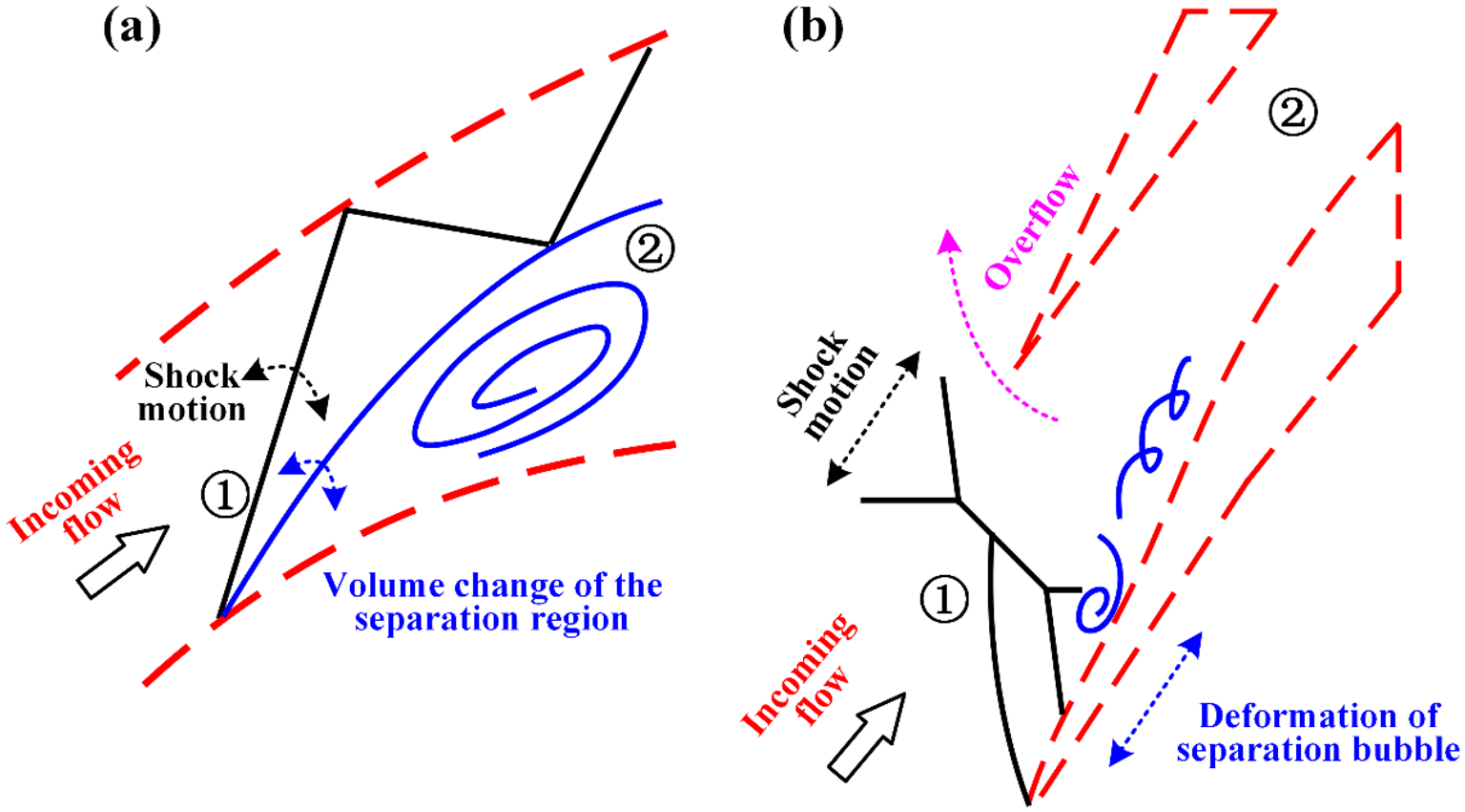
Schematic diagram of flow field evolution during (**a**) separated flow and (**b**) oscillatory flow.

Schematic diagrams of flow field evolution during separated and oscillatory flows are shown in Fig. 16. In summary, the separated flow is dominated by the shock motion. The oscillatory flow is dominated by the shock motion and overflow. Due to the addition of overflow, the shock oscillation is enhanced, and the pressure fluctuates greatly.

## Conclusions

In this study, a detailed analysis of the throttling process in a supersonic cascade was conducted through wind tunnel experiments. Cold flow analysis was performed to study the flow field states at different backpressures. The incoming flow, with a Mach number of 2.4, was choked in a linear manner with the aid of a flap at the exit. The high-frequency wall static pressure and high-speed schlieren images were recorded simultaneously.

The flow field evolution was first analyzed based on the pressure data. During the attached flow, the fluctuation in pressure caused by the impingement of background waves had an energy content in the low-frequency shock oscillation frequency range of 4–550 Hz. During the separated flow, when the transducer on the pressure surface located in the shock train flow was swept by the STLS, the pressure fluctuation energy in the frequency range of 6–600 Hz suddenly increased. With the increase in backpressure, the pressure fluctuation energy in the frequency range of 40–400 Hz on the suction surface located in the subsonic separated flow gradually increased. During the oscillatory flow, when the STLS was detached from the suction surface tip, the pressure of the entire cascade channel fluctuated significantly at a frequency of 52.49 Hz.

The flow field evolution was then analyzed based on schlieren images. During the separated flow, the three structures in the shock train, namely the STLS, separated shear layer, and reflected shock waves, were the dominant oscillation structures, and the oscillations of these three structures exhibited a strong coupled relationship. The phase results indicated that the pressure change in the subsonic separated region first led to a change in the state of the separated shear layer, after which the shock wave structures in the shock train, including the STLS and the reflected shock waves, moved. During the oscillatory flow, when the STLS was detached from the suction surface tip, the fluctuations occurring throughout the cascade channel were highly coupled. The phase results indicated that the upstream oscillations of the STLS occurred first, leading to the subsequent fluctuation in the downstream cascade channel.

## Supplementary Information

Supplementary Information.

## Data Availability

The datasets generated during and/or analyzed during the current study are available from the corresponding author on reasonable request.
