# Supplementary material for: Throttling process of a supersonic cascade studied by high-frequency response pressure and high-speed schlieren
Source: Sci Rep. 2021 Jun 30;11:13550. doi: 10.1038/s41598-021-93021-1 (PMC8245612; doi:10.1038/s41598-021-93021-1)
Supplement: Supplementary file 1 — Supplementary Information. [file 41598_2021_93021_MOESM1_ESM.docx]

# Supplementary information: Verification of interpolation methods

The purpose of supplementary information is to verify the interpolation methods in Figs. 7, 8 and 9 in section Ⅲ-A-3. Figs. 7, 8 and 9 are drawn to clarify the spatial distribution of the pressure fluctuation. The points in the figures represent the energy of the pressure fluctuation with a certain frequency at a certain position. However, in this experiment, five transducers are arranged on the suction surface (S1 – S5) and pressure surface (P1 – P5), respectively. Pressure fluctuations at other positions are not clear. In order to draw a complete contour, the pressure fluctuations at other positions are estimated by interpolation method.

As shown in Fig. S1, when the interpolation is not used, the frequency-energy distribution of pressure fluctuation at a certain position is not well displayed. The purpose of interpolation is to better display the frequency-energy distribution of pressure fluctuations at different positions. It is important to note, however, that the true frequency-energy distribution cannot be misled by the interpolation method used. The results of linear interpolation, Pchip interpolation, and Spline interpolation are shown in Figs. S1(b), S1(c), and S1(d). The Pchip interpolation is conformal piecewise cubic interpolation. The values inserted at the query point are piecewise cubic conformal interpolation based on the values at the grid points of adjacent points. The Spline interpolation uses non-junction termination conditions. The values inserted at the query points are cubic interpolation based on the values at the grid points of adjacent points in each dimension. As shown in Figs. S1(c) and S1(d), deviations appear in the estimation of pressure fluctuation at *x* < 20 mm by Pchip and Spline interpolations. As shown in Fig. S1(b), the linear interpolation can better display the spatial distribution of pressure fluctuation without misleading the real results. Therefore, linear interpolation is used in this study to draw the spatial distribution contours of pressure fluctuation.


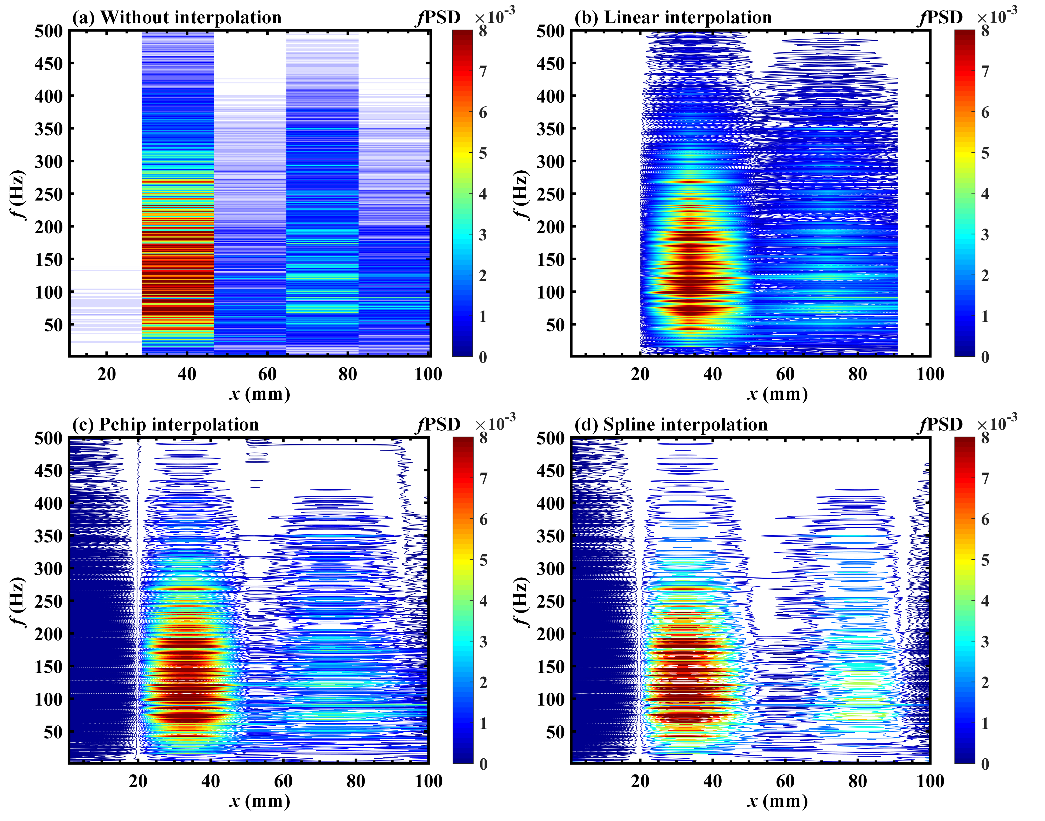


**Figure S1.** Contours of *f*PSD at the suction surface in the separated flow during the period 11.554 s < *t* < 13.989 s calculated **(a)** without interpolation, **(b)** by linear interpolation, **(c)** by Pchip interpolation, and **(d)** Spline interpolation.
